# Mitral Annular Function in Mitral Annular Calcification and Severe Mitral Regurgitation

**DOI:** 10.1016/j.jacadv.2025.102161

**Published:** 2025-09-17

**Authors:** Joseph Kassab, Joseph Hajj, Serge C. Harb, Rhonda Miyasaka, Grant Reed, Amar Krishnaswamy, Kari Feldt, Christopher U. Meduri, A. Marc Gillinov, Shinya Unai, James J. Yun, Marcus Carlsson, Samir R. Kapadia, Rishi Puri

**Affiliations:** a Heart, Vascular and Thoracic Institute, Cleveland Clinic Foundation, Cleveland, Ohio, USA; b Department of Internal Medicine, UT Southwestern Medical Center, Dallas, Texas, USA; c Department of Cardiology, Karolinska University Hospital, Department of Medicine Solna, Karolinska Institute, Stockholm, Sweden

**Keywords:** mitral annular calcification, mitral annular function, mitral regurgitation, transcatheter mitral valve replacement

## Abstract

**Background:**

Longitudinal left ventricular (LV) shortening, or mitral atrioventricular plane displacement (AVPD), reflects mitral annular function.

**Objectives:**

The authors sought to demonstrate the use of cardiac computed tomography (CCT) to measure mitral AVPD and assess its contribution to LV stroke volume (LVSV) in patients with mitral annular calcification (MAC), severe functional mitral regurgitation (FMR), and severe primary mitral regurgitation (PMR) without MAC.

**Methods:**

We included 200 patients with circumferential MAC (age 79.6 ± 10 years), 50 with severe FMR (age 74 ± 8 years), 50 with severe PMR (age 83 ± 10 years), and 50 control subjects (age 41.6 ± 16 years) who underwent CCT (2016-2022). AVPD was measured in all patients. The volume of blood attributable to AVPD (V^AVPD^) and its fractional contribution to LVSV (SV^AVPD%^) were calculated across all cohorts. Group comparisons were performed using *t*-tests, analysis of variance, and Chi-square tests; multivariable linear regression was used to identify independent associations with AVPD and SV^AVPD%^.

**Results:**

Mean AVPD differed significantly between controls, PMR, and FMR patients (11.9, 6.6, 9.9 mm; *P* < 0.0001), whereas SV^AVPD%^ was similar (45.8%, 46.5%, 45.5%; *P* = 0.94). Among MAC patients, AVPD decreased with increasing severity (*P* < 0.0001). Those with grade 3 to 4 MAC had significantly lower SV^AVPD%^ compared to non-MAC groups (26.7% to 28.9% vs 45.5% to 46.5%; *P* < 0.0001). MAC severity was independently associated with reduced AVPD and SV^AVPD%^ (*P* < 0.0001).

**Conclusions:**

CCT can quantify mitral annular function and its contribution to LVSV. Moderate-to-severe MAC significantly impairs annular function, reducing its share of LVSV. These findings may inform patient selection for transcatheter mitral therapies that impact annular dynamics.

Longitudinal left ventricular (LV) shortening, also known as atrioventricular plane displacement (AVPD), represents a piston-like “pumping” movement of the base of the ventricles toward the apex during the cardiac cycle, which essentially represents the annular function of the atrioventricular valves.^1^ Prior studies have shown cardiac magnetic resonance imaging (CMR), but not echocardiography, demonstrate the true extent of mitral AVPD which accounts for a significant proportion (50% to 60%) of LV stroke volume (SV) in athletes, healthy controls, and systolic heart failure patients—irrespective of LV ejection fraction (LVEF).^2^ Echocardiography underestimates AVPD due to limited spatial resolution, off-axis image acquisition, and variability in annular tracking, which reduces its accuracy in assessing subtle longitudinal motion. Cardiac computed tomography (CCT) represents the most commonly used imaging modality following echocardiography when screening patients for various transcatheter mitral valve therapies,^3^ yet no formal validation currently exists using CT to assess mitral annular function. Although CMR is a robust modality for quantifying AVPD, it is not routinely used in the preprocedural workflow for transcatheter mitral valve replacement (TMVR) due to limited availability, longer acquisition times, patient-related constraints (eg, arrhythmia, implanted devices, and claustrophobia), and lack of integration into existing TMVR planning protocols.

Various TMVR platforms are currently in different stages of development with the aim to completely eliminate mitral regurgitation (MR), which can be challenging with mitral transcatheter edge-to-edge-repair (M-TEER) technologies, especially in complex mitral valve disease or functional MR (FMR). However, the fact that M-TEER preserves annular function represents an advantage, with the surgical literature demonstrating significant acute declines in LVEF following surgical mitral valve replacement.^4^^,^^5^ Mitral annular calcification (MAC) represents high-risk mitral anatomy, often considered a poor anatomical substrate for M-TEER devices.^6^ However, from a physiological standpoint, MAC may precondition the mitral annulus to contribute significantly less to mitral annular function, and thus LV SV, which mechanistically may represent a better anatomic substrate for TMVR devices that require annular/subannular fixation.^7^ However, mitral annular function in the setting of MAC and its contribution to LV SV has not been described. The present study was thus aimed to validate the use of CCT to measure mitral AVPD, a metric for mitral annular function, and subsequently explore its contribution toward overall LV SV in patients with varying degrees of MAC, severe FMR, and severe primary (or degenerative) MR (PMR).

## Materials and methods

### Study population

This study was approved by the Cleveland Clinic Institutional Review Board, received proper ethical oversight, and was deemed an exempt protocol, not requiring written consent. From January 2016 to January 2022, patients (cases) with various degrees of circumferential MAC, patients with isolated severe FMR (with minimal-to-no MAC), patients with isolated severe PMR (with minimal-to-no MAC), and control subjects were identified from an all-comer cohort of patients referred for CCT at the Cleveland Clinic. The group sizes were selected to provide balanced representation of the phenotypes of interest while keeping the data set manageable for detailed manual image analysis.

Severe FMR or PMR was defined as per the established guidelines including an Effective Regurgitant Orifice Area ≥0.40 cm^2^ and a regurgitant volume ≥60 mL.^8^

In addition to quantitative thresholds, severity was supported by the presence of a prominent central or eccentric regurgitant jet on color Doppler, dense and triangular continuous wave Doppler signal, and systolic flow reversal in pulmonary veins when available.

For FMR, patients demonstrated structurally normal mitral leaflets with restricted motion due to underlying LV remodeling, whereas PMR cases exhibited degenerative changes such as prolapse or flail leaflets. Patients with multiphasic CCT were included in the study. Controls comprised patients without any valvular dysfunction and normal coronary CT. All included FMR patients were defined as having ventricular FMR.

### CCT acquisition protocol and image analysis

From January 2016 to January 2022, noncoronary dedicated multiphasic CCT scanning protocols remained similar. Multidetector CT technology (Philips iCT 256-slice scanner; Siemens Definition Force Dual Source Scanner; Siemens Somatom Force Dual Source Scanner) was employed. Spiral imaging with retrospective electrocardiogram gating, thin-sliced acquisition (0.9-mm slice reconstruction) was performed of the chest (including the entire heart from the superior vena cava to the suprahepatic inferior vena cava), with 3-dimensional (3D) and dynamic 4D assessment. Intravenous (IV) administration of 60 mL to 140 mL of low-osmolar contrast material (Omnipaque 350, GE Healthcare) was used for each patient via an 18-gauge IV needle in an antecubital vein. The CT studies were performed without electrocardiogram dose modulation, using retrospective gating. Full-dose mAs was applied throughout the cardiac cycle, including end-systole and end-diastole. Tube voltage (kVp) was set at 120 for patients weighing ≥280 pounds and 100 for patients weighing <280 pounds. Subsequently, nongated flash mode spiral imaging (1- and 3-mm slice reconstruction) of the chest, abdomen, and pelvis was performed without additional IV administration of contrast material. For optimization of anatomical evaluation, multiplanar reconstruction, maximum intensity projections, and advanced 3D and 4D offline postprocessing were performed on a dedicated stand-alone workstation. Reconstructions were performed at every 5 or 10% of the R-R interval covering the entire cardiac cycle (full R-R). All CCT images were analyzed on an Aquarius workstation (TeraRecon).

### SV calculation

LV SV calculation using multiphasic CCT was performed. Endocardial borders of the LV were delineated for both systolic and diastolic phases. The software applied the Simpson method, a technique that involves calculating the volume of each slice of the left ventricle by summing the volumes of a series of discs.^9^ These discs are obtained by dividing the ventricle into parallel, equally spaced slices. The SV was determined by subtracting the end-systolic volume from the end-diastolic volume. Papillary muscles and trabeculations were included as part of the LV cavity during SV calculation, following the default settings and volumetric analysis capabilities of the TeraRecon Aquarius software.

### Measurement of AVPD, volume of blood attributable to avpd, And percentage contribution of AVPD to SV

**Figure 1:**
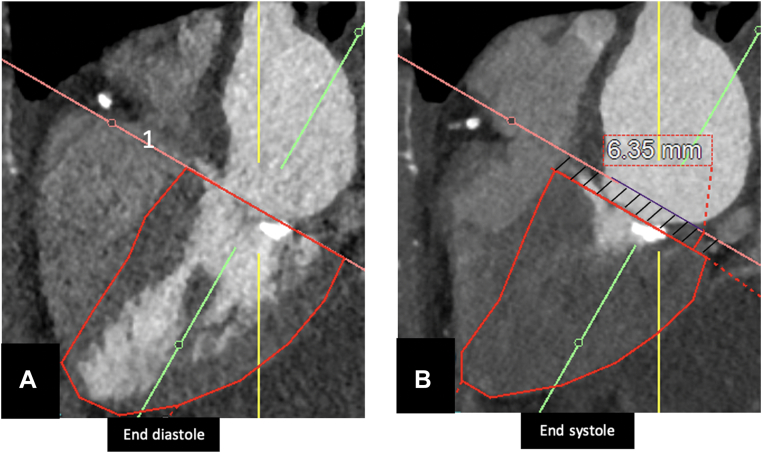
**Measurement of Mitral AVPD Using CCT on a 4-Chamber View** The AVPD can be seen as a piston-like movement of the AV plane from the base to the apex within the LV. On CCT, AVPD was measured in three long-axis images (4-chamber view, 2-chamber view, and LV outflow track view). The positions of the mitral AV plane in end diastole (A) and in end systole (B) were determined in the 3 views, with the vertical down placement of the MV plane from diastole to systole corresponding to the AVPD height (in mm). The average of the three locations was calculated. AVPD = atrioventricular plane displacement; CCT = cardiac computed tomography.

**Figure 2:**
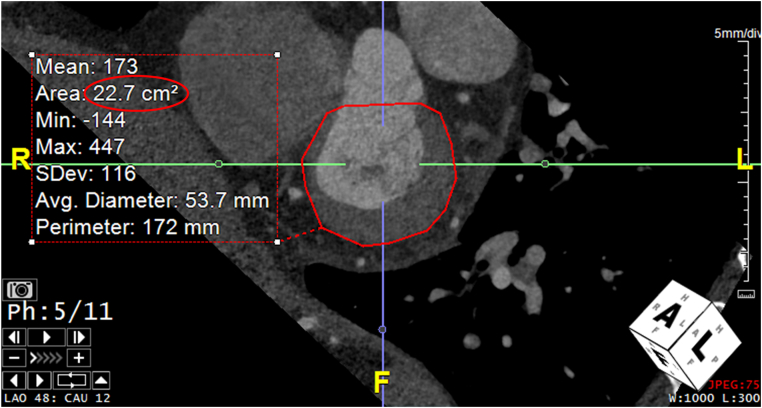
Measurement of LV Short Axis Area using Cardiac CT The volume of pumped blood generated by the AVPD (VAVPD) was calculated as the LV short-axis area multiplied by the AVPD height. As demonstrated by Carlsson et al^2^ and validated for cardiac MRI, the area multiplied by the AVPD cannot be the short-axis area at the mitral annulus but rather must be the largest epicardial short-axis area of the LV as it is constant throughout the cardiac cycle. LV = left ventricular; other abbreviations as in Figure 1.

The AVPD can be visualized as a piston-like movement of the AV plane from the base to the apex within the LV.^2^ Reproducing the validated MRI technique by Carlsson et al^2^ on CCT, AVPD was measured in three long-axis images (4-chamber view, 2-chamber view, and LV outflow tract view). The positions of the mitral AV plane in end-diastole and in end-systole were determined in the three views, with the vertical down placement of the mitral valve plane from diastole to systole corresponding to the AVPD height (in mm) (Figures 1 and 2). The average of the three locations was calculated. Subsequently, the volume explained by the AVPD (V^AVPD^) (in mL) was calculated by multiplying the LV short axis area by the AVPD. Finally, the contribution of V^AVPD^ to the SV (SV^AVPD%^) was calculated by dividing V^AVPD^ by the SV calculated on CCT (%).

### MAC severity grading

**Figure 3:**
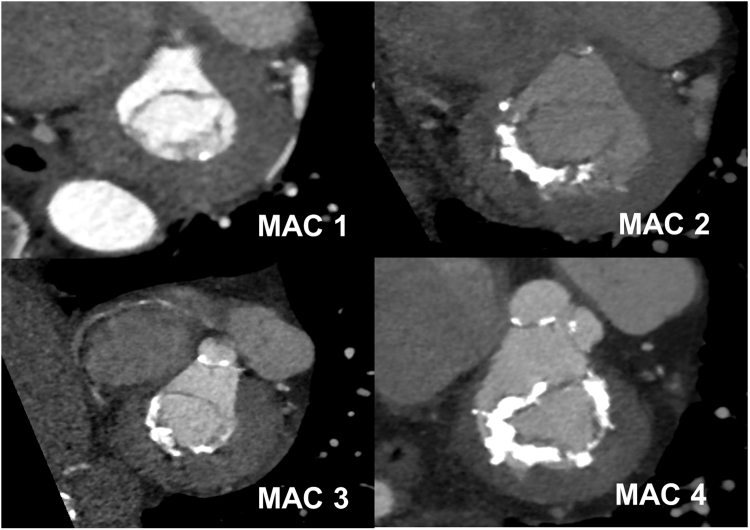
**Stratification of Circumferential MAC Severity** The severity of circumferential MAC was assessed via CCT. Patients were stratified into 4 distinct groups corresponding to the extent of calcium distribution around the annulus circumference (expressed in degrees of circumference involved). The groups were defined as follows: MAC 1 (calcifications <90°), MAC 2 (90-180°), MAC 3 (181-270°), and MAC 4 (>270°). MAC = mitral annular calcification.

The severity of circumferential MAC was assessed via CCT using a simplified method based on the one described by Guerrero et al.^10^ Patients were stratified into 4 distinct groups corresponding to the extent of calcium distribution around the annulus circumference (expressed in degrees of circumference involved). The groups were defined as follows: MAC 1 (calcification <90°), MAC 2 (90-180°), MAC 3 (181-270°), and MAC 4 (>270°) (Figure 3).

SV, AVPD height, V^AVPD^, and SV^AVPD%^ measurements and calculations were performed on subjects with FMR, PMR, and MAC, as well as controls without any valvular disorder.

### Practical examples

To illustrate the clinical implications of our findings, we sought to identify patients who underwent both premitral and postmitral intervention CCT during the study period. Due to the rarity of such paired scans in routine practice, except as part of a prospective research protocol, only three cases were identified. We measured their AVPD with subsequent contribution of mitral annular function to the SV preintervention and postintervention (SV^AVPD%^). These illustrative cases were not intended for statistical inference, but rather to serve as real-world vignettes complementing the overall cohort analysis.

### Statistical analysis

Categorical variables are reported as frequencies and percentages and compared using the Chi-square test. Numeric variables are reported as mean ± SD and compared using the Student's t-test if normally distributed or reported as median (IQR) and compared using the Mann-Whitney test if not normally distributed. In cases where more than 2 groups were compared, analysis of variance was employed. Measurements of volumes and distances are expressed as means ± SD. The relationship between variables was determined by the Pearson correlation coefficient. To study variables independently associated with AVPD height change, linear regression and multivariate analysis were performed. All models included the following covariates based on clinical relevance and prior literature: age, sex, body mass index, LVEF, NYHA functional class, atrial fibrillation, MAC severity, and grades of concomitant valvular heart disease (aortic stenosis [AS], aortic regurgitation [AR], MR, tricuspid regurgitation [TR]), and right ventricular function. The Shapiro-Wilk test was used to assess the normality of AVPD and SV^AVPD%^. To assess the reproducibility of the novel AVPD parameter, we performed a Bland-Altman analysis of both intraobserver and interobserver variability. A Pearson correlation coefficient was also computed to evaluate the linear relationship between the 2 sets of intraobserver measurements. We calculated the mean difference ±SD of the paired measurements. A value of *P* < 0.05 was defined as statistically significant. All statistical analyses were performed using R (version 4.3.1) using publically available packages and IBM SPSS Statistics (version 29).

## Results

### Study population

300 cases and 50 controls were included from an all-comer cohort of consecutive CCT referrals at a quaternary/tertiary care CCT lab: 200 patients with varying degrees of circumferential MAC (mean age 79.6 ± 10 years), 50 patients with severe FMR (mean age 74 ± 8 years), 50 patients with severe PMR (mean age 83 ± 10 years), and 50 control subjects (MAC 0) (mean age 41.6 ± 16 years). Baseline patient characteristics are listed in Table 1 and transthoracic echocardiography characteristics of the cohort are listed in Tables 2 and 3. The median time difference between echocardiogram and CCT was 24 days (IQR: 0-56 days). Shapiro-Wilk testing demonstrated that both AVPD and SV^AVPD%^ were normally distributed across the data set (*P* > 0.05 for each in the overall cohort). There were no missing data for any of the categorical or continuous variables included in the analyses.

**Table 1:** Patient Characteristics Clinical and Demographic Characteristics

	Controls (n = 50)	FMR (n = 50)	PMR (n = 50)	MAC (1-4) (n = 200)	*P* Value^a^
Age, y	41.6 ± 16	74 ± 8	83 ±10	79.6 ± 10	<0.001
Females, n (%)	22 (44)	20 (40)	22 (44)	122 (61)	0.008
BMI, kg/m^2^	31.5 ± 8.1	32.5 ± 6.5	25.6 ± 3.9	29.9 ± 7.4	<0.001
Atrial fibrillation, n (%)	4 (8)	22 (44)	11 (22)	73 (36.5)	<0.001
Hypertension, n (%)	26 (52)	38 (76)	15 (30)	146 (73)	<0.001
Dyslipidemia, n (%)	8 (16)	36 (72)	15 (30)	107 (53.5)	<0.001
Diabetes, n (%)	4 (8)	14 (28)	10 (20)	68 (34)	0.001
Coronary artery disease^b^, n (%)	0 (0)	34 (68)	21 (42)	100 (50)	<0.001
Chronic kidney disease^c^, n (%)	0 (0)	4 (8)	5 (10)	42 (21)	<0.001
LVEF, %	54 ± 6.9	36 ± 12.9	64 ± 5.5	60 ± 9.7	<0.001
NYHA functional class					
I	48 (96)	1 (2)	10 (20)	13 (6.5)	<0.001
II	2 (4)	18 (36)	22 (44)	57 (28.5)	<0.001
III	0 (0)	30 (60)	14 (28)	125 (62.5)	<0.001
IV	0 (0)	1 (2)	4 (8)	3 (1.5)	0.07
ACEi/ARB, n (%)	10 (20)	40 (80)	20 (40)	120 (60)	<0.001
Beta-blocker, n (%)	8 (16)	45 (90)	18 (36)	130 (65)	<0.001
Diuretics, n (%)	5 (10)	44 (88)	25 (50)	110 (55)	<0.001
Digoxin, n (%)	1 (2)	10 (20)	5 (10)	30 (15)	0.002

ACEi/ARB = angiotensin-converting enzyme inhibitors/angiotensin II receptor blockers; BMI = body mass index; B-blocker = beta-blockers; FMR = functional mitral regurgitation; LVEF = left ventricular ejection fraction; MAC = mitral annular calcification; PMR = primary mitral regurgitation.

a *P* values for categorical variables were calculated using Chi-square tests and for continuous variables were calculated using ANOVA.

b History of acute coronary syndrome, stable angina, or one or more stenotic coronary artery.

c Defined as presence of kidney damage or an estimated glomerular filtration rate (eGFR) <60 mL/min/1.73 m^2^, persisting for 3 months or more, irrespective of the cause.

**Table 2:** Transthoracic Echocardiography Characteristics in the MAC Cohort

	MAC 1 (n = 50)	MAC 2(n = 50)	MAC 3(n = 50)	MAC 4(n = 50)	*P* Value^a^	Total (N = 200)
LVEDV, mL	95.6 ± 32.4	94.6 ± 46	91 ± 43	88 ± 31	0.70	92 ± 39
LVESV, mL	39.4 ± 21.6	40.6 ± 37	37 ± 25	38 ± 21	0.90	38 ± 27
LVEF, %	60.7 ± 9.1	59 ± 12	62 ± 7.7	59 ± 10	0.34	60 ± 9.7
LV mass index, g/m^2^	99.1 ± 27	103 ± 36	110 ±37	111 ±29	0.27	108 ± 46
LA volume index, mL/m^2^	43.9 ± 19.3	41.7 ± 17	48.1 ± 10	58 ±18	<0.001	48 ± 17
Mitral stenosis^b^, n (%)					0.15	
None	33 (66)	40 (80)	38 (76)	27 (54)		138 (69)
Mild	11 (22)	6 (12)	4 (8)	12 (24)		33 (16.5)
Moderate	3 (6)	3 (6)	7 (14)	11 (22)		24 (12)
Severe	3 (6)	1 (2)	1 (2)	0 (0)		5 (2.5)
Mitral regurgitation, n (%)					0.22	
None	10 (20)	4 (8)	7 (14)	3 (6)		24 (12)
Grade I (mild)	22 (44)	32 (64)	20 (40)	30 (60)		104 (52)
Grade II (mild-to-moderate)	11 (22)	12 (24)	17 (34)	10 (20)		50 (25)
Grade III (moderate-to-severe)	5 (10)	2 (4)	5 (10)	5 (10)		17 (8.5)
Grade IV (severe)	2 (4)	0 (0)	1 (2)	2 (4)		5 (2.5)
Peak MV gradient, mm Hg	10.5 ± 4.9	9.7 ± 3.2	11.8 ± 4.2	12.7 ± 4.6	0.006	11.4 ± 4.1
Mean MV gradient, mm Hg	4.2 ± 2.3	4 ± 1.85	4.8 ± 2.2	5.2 ± 2.2	0.037	4.7 ± 2.1
E velocity, m/s	0.93 ± 0.28	1.08 ± 0.3	1.33 ± 0.3	1.40 ± 0.4	<0.001	1.2 ± 0.4
A velocity, m/s	1.04 ± 0.32	1.10 ± 0.4	1.16 ± 0.5	1.28 ± 0.5	0.13	1.15 ± 0.4
E/A	1.12 ± 0.89	1.82 ± 3.8	1.29 ± 0.76	2.0 ± 4.5	0.54	1.57 ± 3.01
E/e’	16.2 ± 6.6	19.1 ± 6.5	23.2 ± 7.7	28.3 ± 11.2	<0.001	21.5 ± 9.2
RV systolic pressure, mm Hg	42.7 ± 17.6	35.9 ± 8.9	40.9 ± 13.3	46 ± 18.3	0.01	41.4 ± 15.2
RV qualitative systolic function, n (%)					0.19	
Normal	40 (80)	39 (78)	46 (92)	45 (90)		170 (85)
Low normal/mildly decreased	9 (18)	9 (18)	3 (6)	4 (8)		25 (12)
Moderately decreased	1 (2)	1 (2)	1 (2)	1 (2)		4 (2)
Severely decreased	0 (0)	1 (2)	0 (0)	0 (0)		1 (0.5)
TAPSE, cm	1.9 ± 0.43	1.8 ± 0.46	1.8 ± 0.46	1.8 ± 0.47	0.97	1.8 ± 0.45
Tricuspid regurgitation, n (%)					0.77	
None	14 (28)	14 (28)	12 (24)	11 (22)		51 (25.5)
Mild	24 (48)	21 (42)	25 (50)	27 (54)		97 (48.5)
Moderate	7 (14)	10 (20)	11 (22)	10 (20)		38 (19)
Severe	5 (10)	5 (10)	2 (4)	2 (4)		14 (7)
Aortic stenosis, n (%)					0.65	
None	9 (18)	5 (10)	1 (2)	7 (14)		22 (11)
Mild	2 (4)	1 (2)	1 (2)	0 (0)		4 (2)
Moderate	6 (12)	9 (18)	10 (20)	2 (4)		27 (13.5)
Severe	33 (66)	35 (70)	38 (76)	41 (82)		147 (73.5)
Aortic regurgitation, n (%)					0.31	
None	20 (40)	19 (38)	17 (34)	21 (42)		77 (38.5)
Grade I (mild)	19 (38)	19 (38)	22 (44)	20 (40)		80 (40)
Grade II (mild-to-moderate)	7 (14)	8 (16)	5 (10)	6 (12)		26 (13)
Grade III (moderate-to-severe)	4 (8)	3 (6)	4 (8)	3 (6)		14 (7)
Grade IV (severe)	0 (0)	1 (2)	2 (4)	0 (0)		3 (1.5)

A velocity = atrial contraction velocity; E velocity = early diastolic filling velocity; E/A = ratio of early to atrial contraction velocity; E/e’ = ratio of early diastolic filling velocity to early diastolic mitral annular velocity; LA = left atrial; LV = left ventricular; LVEDV = left ventricular end-diastolic volume; LVESV = left ventricular end-systolic volume; MV = mitral valve; RV = right ventricular; TASPE = tricuspid annular plane systolic excursion; other abbreviations as in Table 1.

a *P* values for categorical variables were calculated using Chi-square tests and for continuous variables were calculated using ANOVA.

b Defined as mild (mitral valve area >1.5 cm^2^, mean mitral valve gradient <5 mm Hg, and pulmonary artery systolic pressure <30 mm Hg), moderate (mitral valve area 1-1.5 cm^2^, mean mitral valve gradient 5-10 mm Hg, and pulmonary artery systolic pressure 30-50 mm Hg), and severe (mitral valve area <1 cm^2^, mean valve gradient >10 mm Hg, and pulmonary artery systolic pressure >50 mm Hg).

**Table 3:** Transthoracic Echocardiography Characteristics in the Study Population

	Controls (n = 50)	FMR (n = 50)	PMR (n = 50)	MAC (1-4) (n = 200)	*P* Value^a^
LVEDV, mL	127 ± 61	172 ± 68	103 ± 32	92 ± 39	<0.001
LVESV, mL	57 ± 36	115 ± 67	40 ± 15	38 ± 27	<0.001
LVEF, %	54 ± 6.9	36 ± 12.9	64 ± 5.5	60 ± 9.7	<0.001
LV mass index, g/m^2^	94 ± 31	132 ± 32	113 ± 31	108 ± 46	<0.001
LA volume index, mL/m^2^	32 ± 15	69 ± 34	68 ± 39	48 ± 17	<0.001
Mitral stenosis^b^, n (%)					
None	50 (100)	50 (100)	50 (100)	138 (69)	<0.001
Mild	0 (0)	0 (0)	0 (0)	33 (16.5)	
Moderate	0 (0)	0 (0)	0 (0)	24 (12)	
Severe	0 (0)	0 (0)	0 (0)	5 (2.5)	
Mitral regurgitation, n (%)					
None	50 (100)	0 (0)	0 (0)	24 (12)	<0.001
Grade I (mild)	0 (0)	0 (0)	0 (0)	104 (52)	
Grade II (mild-to-moderate)	0 (0)	0 (0)	0 (0)	50 (25)	
Grade III (moderate-to-severe)	0 (0)	0 (0)	0 (0)	17 (8.5)	
Grade IV (severe)	0 (0)	50 (100)	50 (100)	5 (2.5)	
Peak MV gradient, mm Hg	-	10 ± 1.4	13.8 ± 5.4	11.4 ± 4.1	<0.001
Mean MV gradient, mm Hg	-	2.75 ± 0.5	4.4 ± 0.9	4.7 ± 2.1	<0.001
E velocity, m/s	0.9 ± 0.3	1.2 +/0.3	1.3 ± 0.43	1.2 ± 0.4	<0.001
A velocity, m/s	0.7 ± 0.3	0.6 ± 0.4	0.87 ± 0.3	1.15 ± 0.4	<0.001
E/A	1.4 ± 0.7	2.5 ± 1.5	1.65 ± 0.8	1.57 ± 3.01	0.07
E/e’	8.8 ± 2.8	21.2 ± 8.5	18.5 ± 7.6	21.5 ± 9.2	<0.001
RV systolic pressure, mm Hg	33 ± 8.8	49 ± 20.4	46.7 ± 13	41.4 ± 15.2	<0.001
RV function, n (%)					
Normal	50 (100)	20 (40)	36 (72)	170 (85)	<0.001
Low normal/mildly decreased	0 (0)	23 (46)	10 (20)	25 (12)	
Moderately decreased	0 (0)	7 (14)	4 (8)	4 (2)	
Severely decreased	0 (0)	0 (0)	0 (0)	1 (0.5)	
TASPE, cm	1.9 ± 0.5	1.8 ± 0.6	2 ± 0.5	1.8 ± 0.45	0.052
Tricuspid regurgitation, n (%)					
None	28 (56)	5 (10)	10 (20)	51 (25.5)	<0.001
Mild	20 (40)	18 (36)	18 (36)	97 (48.5)	
Moderate	2 (4)	20 (40)	12 (24)	38 (19)	
Severe	0 (0)	7 (14)	10 (2)	14 (7)	
Aortic stenosis, n (%)					
None	46 (92)	41 (82)	44 (88)	22 (11)	<0.001
Mild	2 (4)	3 (6)	2 (4)	4 (2)	
Moderate	2 (4)	3 (6)	2 (4)	27 (13.5)	
Severe	0 (0)	3 (6)	2 (4)	147 (73.5)	
Aortic regurgitation, n (%)					
None	40 (80)	25 (50)	40 (80)	77 (38.5)	<0.001
Grade I (mild)	8 (16)	11 (22)	8 (16)	80 (40)	
Grade II (mild-to-moderate)	1 (2)	6 (12)	1 (2)	26 (13)	
Grade III (moderate-to-severe)	1 (2)	5 (10)	1 (2)	14 (7)	
Grade IV (severe)	0 (0)	3 (6)	0 (0)	3 (1.5)	

Abbreviations as in Tables 1 and 2.

a *P* values for categorical variables were calculated using chi-square tests and for continuous variables were calculated using ANOVA.

b Defined as mild (mitral valve area >1.5 cm^2^, mean mitral valve gradient <5 mm Hg, and pulmonary artery systolic pressure <30 mm Hg), moderate (mitral valve area 1-1.5 cm^2^, mean mitral valve gradient 5-10 mm Hg, and pulmonary artery systolic pressure 30-50 mm Hg), and severe (mitral valve area <1 cm^2^, mean valve gradient >10 mm Hg, and pulmonary artery systolic pressure >50 mm Hg).

### Controls, FMR, and PMR patients

Table 4 describes the AVPD, SV, and SV^AVPD%^ across healthy controls, patients with severe PMR and severe FMR. The average AVPD in controls was 11.9 ± 1.01 mm, with the mean SV^AVPD%^ being 45.8% ± 11.6%. In those with severe FMR, the AVPD was 6.6 ± 0.75 mm, with the SV^AVPD%^ being 46.5% ± 10.6%. In the severe PMR population, the AVPD was 9.9 ± 1.30 mm, with the SV^AVPD%^ being 45.5% ± 10.7%. Although the AVPD differed significantly in the healthy controls vs patients with severe PMR or FMR (*P* < 0.0001), the SV^AVPD%^ did not significantly differ across these 3 groups (*P* = 0.94), suggesting that the proportional contribution of mitral annular function to the LV SV did not differ across these 3 fundamentally different groups of patients with noncalcific mitral annuli.

**Table 4:** AVPD, LVSV, Volume (of SV) Generated by AVPD, and the Fraction of the LV SV Attributable to AVPD Across Varying Degrees of MAC, FMR, and PMR as Compared to Healthy Controls

	Controls (n = 50)	FMR (n = 50)	PMR (n = 50)	*P* Value	MAC 1 (n = 50)	MAC 2 (n = 50)	MAC 3 (n = 50)	MAC 4 (n = 50)	*P* Value MAC 1-4
AVPD, mean mm (SD)	11.9 (1.02)	6.6 (0.75)	9.9 (1.3)	<0.0001	8.8 (1.02)	7.5 (0.4)	6.6 (0.6)	5.3 (0.7)	<0.0001
V^AVPD^, mean mL (SD)	33.6 (8.2)	26.4 (5.1)	27.9 (7.1)	0.001	22.9 (5.8)	19.5 (4.4)	16.9 (3.0)	13.9 (3.1)	<0.0001
SV, mean mL (SD)	79.2 (18.2)	59.8 (17.9)	63.6 (18.7)	<0.01	64.3 (19.8)	58.3 (17.3)	58.4 (21.8)	51.9 (14.1)	0.01
SV^AVPD%^ mean % (SD)	45.8 (11.6)	46.5 (10.6)	45.5 (10.7)	0.943	35.6 (6.7)	33.4 (7.4)	28.9 (8.1)	26.7 (8.5)	<0.0001

AVPD = atrioventricular plane displacement; SV = troke volume; SV^AVPD%^ = contribution of V^AVPD^ to the SV; V^AVPD^ = volume of pumped blood generated by the AVPD; other abbreviation as in Tables 1 and 2.

### MAC patients

**Figure 4:**
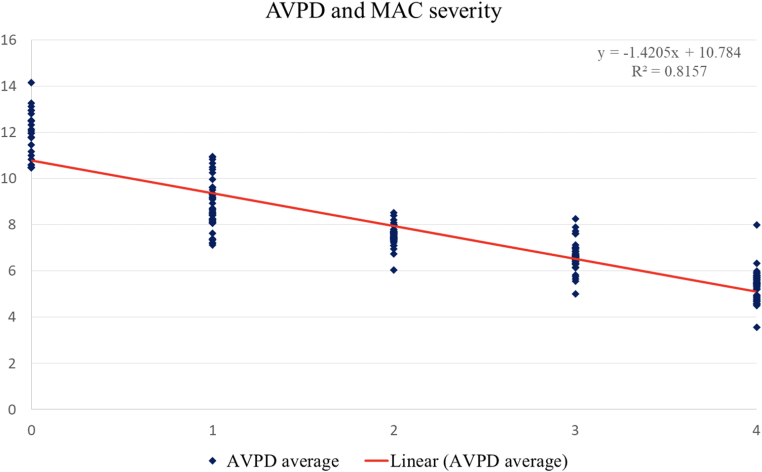
**Correlation Analysis of AVPD (mm) and MAC Severity** Following Pearson correlation coefficient calculation in MAC patients (n = 200), a strong negative correlation was found between MAC severity (0-4) and AVPD (mitral annular function) (R = −0.903). Abbreviation as in Figures 1 and 3.

Table 5 also describes the AVPD and the SV^AVPD%^ according to the presence and degree of MAC. In patients with MAC (grades 1-4), the average AVPD significantly decreased in a stepwise fashion in line with increasing MAC severity from 8.8 ± 1.02 mm in patients with MAC 1 down to 5.3 ± 0.7 mm in patients with MAC 4 (*P*_trend across MAC groups_ <0.0001). The Pearson correlation coefficient calculation between MAC severity and AVPD is shown in Figure 4 (R = −0.903) (*P* < 0.0001). Compared to patients with noncalcific mitral annuli (healthy controls, severe PMR, and severe FMR), the presence of any MAC conferred a significant reduction in AVPD (*P* < 0.0001) (Table 5). In addition, those with grade 3 to 4 MAC demonstrated the lowest AVPD heights compared to patients with noncalcific annuli (*P* < 0.0001). Moreover, the SV^AVPD%^ in MAC patients was significantly less compared to those with noncalcific mitral annuli (*P* < 0.0001). Greater MAC severity was significantly associated with decreasing SV^AVPD%^ (*P*_trend across MAC groups_ <0.0001). The greatest degrees of MAC (grades 3-4) demonstrated the lowest SV^AVPD%^ compared with those with noncalcific mitral annuli (*P* < 0.0001) (Table 5).

**Table 5:** Comparative Analysis of AVPD and Its Contribution to the LV SV Across MAC, FMR, and PMR Groups

Measurement	FMR (n = 50)	PMR (n = 50)	MAC (1-4) (n = 200)	*P* Value	MAC (3-4) (n = 100)	*P* Value
AVPD, mean mm (SD)	6.60 (0.75)	9.90 (1.3)	7.09 (1.5)	<0.0001	5.99 (0.89)	<0.0001
V^AVPD^, mean mL (SD)	26.4 (5.1)	27.9 (7.1)	18.3 (5.4)	<0.0001	15.4 (3.4)	<0.0001
SV, mean mL (SD)	59.8 (17.9)	63.6 (18.7)	58.2 (18.9)	0.39	55.2 (18.5)	0.09
SV^AVPD%^ mean % (SD)	46.5 (10.6)	45.5 (10.7)	32.8 (8.35)	<0.0001	29.7 (8.44)	<0.0001

Abbreviation as in Tables 1, 2, and 4.

Tables 6 and 7 describe multivariable linear regression analyses of factors associated with reduced AVPD and SV^AVPD%^ in MAC patients, respectively. The results demonstrated that both AVPD and SV AVPD were approximately normally distributed across the data set. After controlling for age, sex, body mass index, LVEF, NYHA functional class, atrial fibrillation, MAC severity, grades of AS, AR, MR, and TR, and right ventricular function, MAC severity remained independently associated with a reduced mitral AVPD (*P* < 0.0001) and reduced SV^AVPD%^ (ie, annular function) (*P* < 0.0001). Notably, MR severity was not significantly associated with changes in AVPD (*P* = 0.25) or SV^AVPD%^ (*P* = 0.765).

**Table 6:** Linear Regression and Multivariate Analysis of Factors Associated With AVPD in MAC patients (N = 200)^a^

	Beta-Coefficient	*P* Value
Age	0.013	0.73
Atrial fibrillation	−0.016	0.66
AR grade	0.049	0.15
AS grade	−0.019	0.58
BMI	−0.004	0.93
Female	−0.022	0.54
LVEF	0.028	0.46
MAC severity	−0.928	**<0.0001**
MR grade	−0.049	0.25
NYHA class	−0.049	0.16
RV function	0.003	0.94
TR grade	0.008	0.84

AR = aortic regurgitation; AS = aortic stenosis; MR = mitral regurgitation; TR = tricuspid regurgitation; other abbreviation as in Tables 1, 2, and 4.

**Bold** indicates a statistically significant *P* value (<0.05). Multivariable linear regression analyses of factors associated with reduced AVPD and in MAC patients. After controlling for multiple factors including age, sex, BMI, LVEF, concomitant valvular heart disease, NYHA class, and atrial fibrillation, MAC severity was independently associated with a reduced mitral AVPD (*P* < 0.0001). Notably, MR severity was not significantly associated with changes in AVPD (*P* = 0.25).

a Dependent variable: AVPD.

**Table 7:** Linear Regression and Multivariate Analysis of Factors Associated With SV^AVPD%^ in MAC Patients (N = 200)^a^

	Beta-Coefficient	*P* Value
Age	0.035	0.731
Atrial fibrillation	−0.030	0.758
AR grade	−0.131	0.150
AS grade	0.032	0.730
BMI	−0.137	0.162
Female	0.283	**0.004**
LVEF	−0.252	**0.014**
MAC severity	−0.479	**<0.0001**
MR grade	0.034	0.764
NYHA class	0.034	0.712
RV function	0.151	0.136
TR grade	−0.027	0.802

Abbreviations as in Tables 1, 2, 4, and 6.

**Bold** indicates a statistically significant *P* value (<0.05). Multivariable linear regression analyses of factors associated with reduced SV^AVPD%^ in MAC patients. After controlling for multiple factors including age, sex, BMI, LVEF, concomitant valvular heart disease, NYHA class, and atrial fibrillation, MAC severity was independently associated with a reduced mitral SV^AVPD%^ (ie, annulus function) (*P* < 0.0001). Notably, MR severity was not significantly associated with changes in SV^AVPD%^ (*P* = 0.765).

a Dependent variable: SV^AVP%^.

Finally, we performed a sensitivity analysis restricting the MAC cohort to patients with none, trace, or mild AS, AR, MR, and TR (N = 34 of 200). In this restricted “pure MAC” group, the correlation between MAC severity (grades 1-4) and AVPD average remained strongly negative (Pearson r = −0.86; *P* < 0.0001).

Notably, to confirm the reproducibility of the AVPD parameter, we performed a Bland-Altman analysis on a cohort of 15 patients, comparing measurements both within 1 advanced cardiac imager and between 2 cardiac imagers. The intraobserver variability demonstrated a mean difference of 0.04 ± 0.17 and a Pearson correlation coefficient of 0.96, indicating excellent reproducibility. The interobserver comparison revealed a mean difference of −0.18 ± 0.45 and a correlation of 0.80, indicative of good agreement between observers. These findings underscore the reliability of AVPD as a continuous variable.

### Practical examples

The results are summarized in Table 8. The first patient underwent TMVR for severe MAC, the second patient underwent TMVR for severe FMR without MAC, and the third patient underwent M-TEER for severe MR.

**Table 8:** Practical Real-World Examples in Various Transcatheter Mitral Interventions

Practical Examples	Age at Intervention (y)	Sex	Ef Preintervention (%)	Ef Postintervention (%)	AVPD Average Preintervention (mm)	AVPD Average Postintervention (mm)	SV^AVPD%^ Preintervention (%)	SV^AVPD%^ Postintervention (%)
Patient 1: TMVR for severe MAC	82	Male	55	57	5.39	5.27	32.86	34.59
Patient 2: TMVR for severe FMR with no MAC	64	Female	55	55	8.02	4.71	44.11	20.73
Patient 3: M-TEER for severe MR	77	Male	34	38	6.82	6.63	51.26	52.10

M-TEER = mitral transcatheter edge-to-edge repair; TVMR = transcatheter mitral valve replacement; other abbreviations as in Tables 1, 4 and 6.

To further our findings, we selected three patients from the Cleveland Clinic database who underwent various mitral interventions and associated preintervention and postintervention 4D-gated cardiac CT scans. We measured their AVPD with subsequent contribution of mitral annulus function to the SV preintervention and postintervention. The results are summarized in Table 8. The first patient (82 years old, male) underwent TMVR for severe MAC, the second patient (64 years old, female) underwent TMVR for severe FMR without MAC, and the third patient (77 years old, male) underwent M-TEER for severe MR.

In the patient with severe MAC, AVPD remained virtually unchanged preintervention to postintervention (5.36 mm vs 5.27 mm), as did the mitral annular contribution to overall SV (SV^AVPD%^: 32.86 vs 34.59%). By contrast, in the patient with severe FMR and no MAC, AVPD decreased by nearly half (8.02 mm vs 4.71 mm), accompanied by a reduction of over 50% in the mitral annular contribution to SV (SV^AVPD%^: 44.11 vs 20.73%). Finally, in the patient who underwent M-TEER (annular sparing technique), neither AVPD (6.82 mm vs 6.63 mm) nor the mitral annulus contribution to SV (SV^AVPD%^: 51.26 vs 52.10%) changed significantly after the procedure.

## Discussion

To the best of our knowledge, the present analysis is the first to use CCT to calculate mitral annular function, and further explore its contribution to forward LV SV across control patients, severe PMR, and severe FMR patients. We extend these observations to the extent and severity of MAC to further define how MAC affects mitral annular function (Central Illustration). Our analysis suggests that mitral annular function, expressed as mitral AVPD, is inversely correlated to the severity of MAC. Furthermore, we show that mitral annular function contributes significantly less to overall LV SV in patients with moderate/severe MAC compared to patients with no/little MAC, irrespective of the presence or degree of MR or LV function. Moreover, we show that mitral annular function is low in patients with both severe ventricular FMR (who essentially were heart failure with reduced ejection fraction [HFrEF] patients) and severe PMR (who had preserved LVEF) but still contributes significantly to LV SV in contrast to patients with severe MAC (likely a heart failure with preserved ejection fraction [HFpEF] patient phenotype) and low annular function. These mechanistic insights may harbor important implications for transcatheter mitral therapies designed to anchor within the mitral annulus vs other therapies that preserve mitral annular function. This may be especially relevant for the prediction of functional outcomes, such as postprocedural LV SV for the broad range of mitral valve phenotypes we are faced with treating in daily clinical practice.

**Central Illustration:**
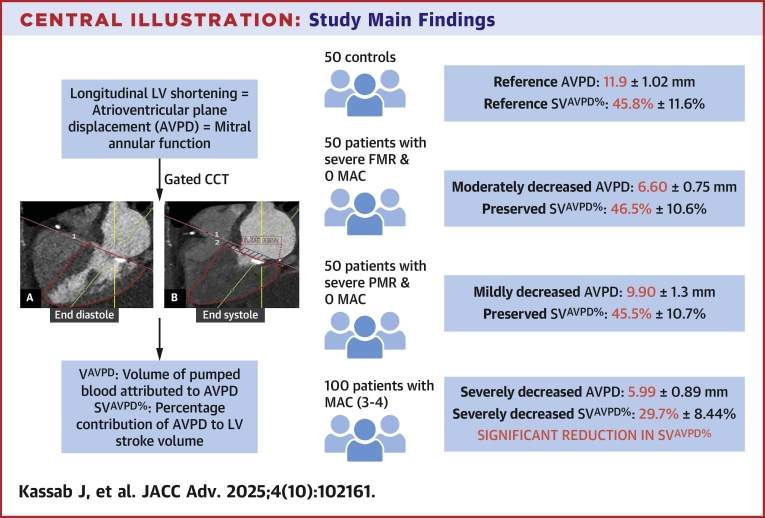
**Study Main Findings** LV longitudinal shortening, measured as AVPD, serves as a surrogate for mitral annular function. In the present study, we validated the use of CCT to assess mitral annular function (AVPD) and quantify the contribution of the mitral annulus to LV stroke volume in controls, patients with increasing degrees of MAC, and patients with severe FMR and PMR without MAC. Our findings demonstrate that mitral annular function significantly contributes to SV in normal circumstances (controls) and in patients with severe FMR and PMR. However, in patients with severe MAC, the contribution of annular function to SV is significantly decreased. This has important implications for patient selection for transcatheter mitral therapies, which may affect mitral annular function to varying degrees, underscoring the importance of assessing mitral annular function in the therapeutic decision-making process. AVPD = atrioventricular plane displacement; CCT = cardiac computed tomography, FMR = functional mitral regurgitation; LV = left ventricular; MAC = mitral annular calcification, PMR = primary mitral regurgitation; SV = stroke volume; SV^AVPD%^ = contribution of V^AVPD^ to the SV; V^AVPD^ = volume of pumped blood generated by the AVPD.

Gated CCT is now established as the primary means of assessing anatomical suitability for a range of structural heart procedures; most notably transcatheter aortic valve replacement, TMVR, and transcatheter tricuspid valve replacement therapies. CCT has primarily focused on assessing valve/conduit structure; with specifics to TMVR including mitral annular size, MAC presence/distribution, and prediction of LV outflow tract obstruction. Although CCT has been useful for TMVR sizing, there is currently no established workflow around understanding mitral annular function, yet mitral annular function contributes 50 to 60% of LV SV,^2^^,^^11^^,^^12^ as measured by the SV^AVPD%^. Although the very initial validation of SV^AVPD%^ occurred using CMR in athlete/patient controls and severe HFrEF patients,^2^ CMR has not emerged as a workhorse imaging modality used for screening patients for transcatheter structural heart therapies. Given the mainstream use of gated CCT workflow for patient screening and procedural planning, the present analysis outlines the opportunity to use CCT imaging to better phenotype potential TMVR candidates according to their mitral structure and annular function, along with the contribution of the mitral annular function to LV SV. Automated calculation of AVPD (and SV^AVPD%^) could provide additional information to optimize the best mitral valve therapeutic strategy for any given patient, many of whom harbor fundamentally different phenotypes (ie, a HFrEF patient with severe FMR and no MAC [who relies on a significant proportion of LV SV derived from mitral annular function] vs a patient with moderate-severe MR/FMR/severe MAC with HFpEF (who relies much less on mitral annular function for LV SV) may not derive the same benefit from a specific type of TMVR therapy]. Given the range of transcatheter therapies now available in feasibility studies that have variable anchoring techniques within the mitral valve and its annulus, left atrium, mitral subapparatus, and coronary sinus,^13^, ^14^, ^15^ their effects on mitral annular function and the net effect on LV SV deserve attention in addition to their MR reducing properties.

The present analysis outlines a conceptual framework (Supplemental Figure 1) to better predict which patients would stand to benefit more from TMVR therapies that rely purely on mitral annular anchoring vs those that reduce MR via different mechanisms.^7^ The surgical literature suggests mitral valve replacement (more than ring annuloplasty) (in addition to other mechanisms such as chordal transection and acute afterload mismatch) significantly reduces mitral annular function and impairs LVEF, with longer-term outcomes worse in those with postoperative LVEF ≤45%.^5^^,^^16^^,^^17^ More recent data using a pure annular-fixating TMVR device demonstrated decreased acute and intermediate LVEF after the TMVR procedure.^18^ The present analysis suggests that greater degrees of MAC significantly lower the mitral annular functional contribution to forward LV SV. Could this mean that severe MAC patients are better suited to purely annular-anchoring TMVR devices? Devices scaffolding the mitral annulus are likely to severely reduce or eliminate its function. However, severe MAC appears to have already preconditioned and inhibited the annular contribution to LV SV by ≈50%. Hence the loss in LV SV from annular fixation is offset by the gains made in MR elimination, with an overall potential favorable net gain in forward LV SV. On the other hand, severe HFrEF patients with zero/little MAC and severe MR might not stand to benefit from annular fixation TMVR devices; rather annular sparing technologies might be preferred in such patients. These patients rely more on mitral annular functional contribution to LV SV, and mitral annular “freezing” or “scaffolding” may reduce LV SV by at least 50%. Unless the regurgitant volume from MR elimination is much larger than the loss of forwards LV SV, the net result in LV SV might not be favorable for the patient. Despite the echocardiogram in these patients showing no/mild residual MR post-TMVR, the thwarted effect on LV SV coupled with abnormal diastolic conditions at the LV base may ultimately result in a lack of clinical/functional improvement in patients. This may be a reason why M-TEER in such patients, despite residual MR, yields favorable clinical and functional outcomes as simply MR reduction (as opposed to total MR elimination) results in a net improvement in LV SV *without* deleterious effects on mitral annular function.

To further illustrate our findings, we identified three patients who underwent different transcatheter mitral interventions and had both preintervention and postintervention gated CCTs available. As shown in the case series, the results align with our overall hypothesis and observations. In the patient who underwent TMVR for severe MAC, mitral annular function did not differ significantly before and after the intervention, although it was initially lower than in controls or in FMR patients without MAC—likely reflecting the already reduced annular contribution in a severely calcified “frozen” mitral annulus. In contrast, the patient with FMR displayed a high baseline mitral annular contribution to SV, which was substantially reduced after TMVR, presumably because the device “freezes” the annulus; this highlights the need to balance any loss of annular function against the benefit of improved forward flow. Finally, in the patient who underwent M-TEER, the nature of the procedure—which clips the leaflets rather than the altering the annulus—did not substantially affect mitral annular function, leaving AVPD and the annular contribution to SV essentially unchanged.

The present data also suggest the importance of each TMVR device to be rigorously evaluated using gated CCT preimplantation and postimplantation to understand its effects on not only mitral annular function, but also the dynamic temporal effects on LV remodeling, LV function, and their relationship to clinical outcomes and quality of life metrics. This may have further implications for early feasibility and pivotal trial strategy for the TMVR landscape as the field is currently grappling with understanding which patient/mitral anatomic phenotype(s) would benefit from which TMVR design.

### Study limitations

Several limitations of the present analysis warrant consideration. The overall sample size (n = 350) appears relatively modest; however, there are a number of differing phenotypic groups evaluated, which adds mechanistic strength to our findings. We do not have gated computed tomography angiography imaging pre-TMVR and post-TMVR or surgical mitral valve replacement (SMVR), hence our hypothesis on what mitral annular scaffolding does to mitral annular function in these patients remains somewhat conjectural. That being said, there is ample evidence that demonstrates impaired LV function in the post-SMVR setting.^19^, ^20^, ^21^ We were unable to differentiate the atrial FMR subtype from the overall FMR population per se, which could demonstrate different annular functional characteristics to those evaluated here. Finally, we acknowledge the potential for selection bias inherent in using a cohort from a single center. This design may limit the generalizability of our findings to broader populations with varying demographic or clinical characteristics. To strengthen the external validity of our results, future multicenter studies would be valuable to validate our results across a wider range of populations and health care settings.

## Conclusions

Routine gated CCT imaging is able to effectively characterize mitral annular function across a range of patient phenotypes, and replicate earlier insights into the relatively equal contribution of mitral annular function to LV SV irrespective of LV function and etiology of severe MR. Large degrees of MAC not only significantly impair mitral annular function but also correspondingly thwart the mitral annular contribution to LV SV. These insights harbor implications for which TMVR therapies might benefit specific patients who present with a range of mitral valve pathologies. Mitral annular functional assessment could become an important additional data point when evaluating patients for TMVR therapies.

## Funding support and author disclosures

The authors have reported that they have no relationships relevant to the contents of this paper to disclose.
