# Supplementary material for: Mitral Annular Function in Mitral Annular Calcification and Severe Mitral Regurgitation
Source: JACC Adv. 2025 Sep 17;4(10):102161. doi: 10.1016/j.jacadv.2025.102161 (PMC12481053; doi:10.1016/j.jacadv.2025.102161)
Supplement: Supplemental Data [file mmc1.docx]

**Supplemental Figure 1**

**Title**: Conceptual framework around an ideal anatomical TMVR candidate from a mitral annular/LV phenotype perspective

**
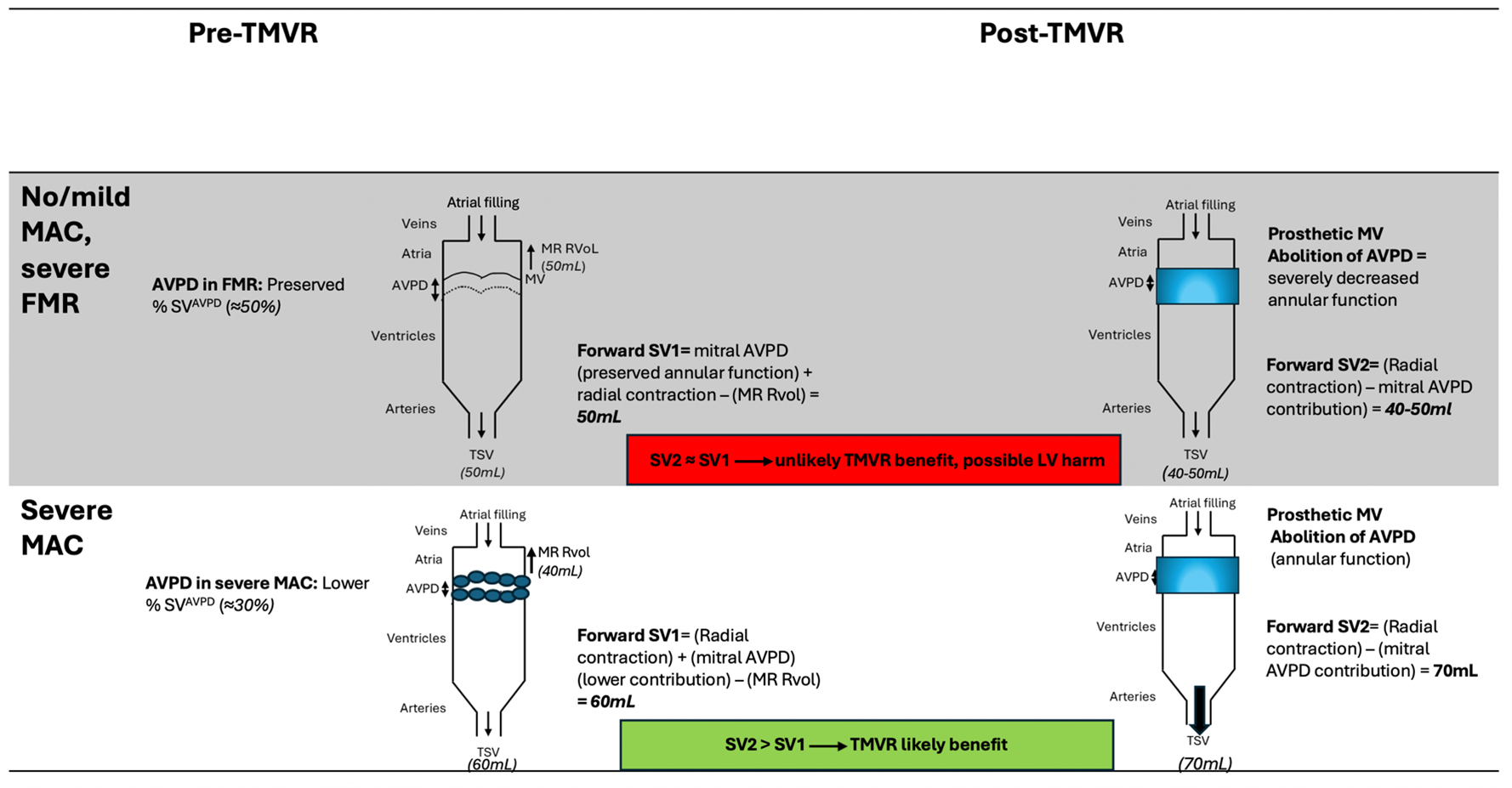
**

**Caption**: The present analysis suggests that greater degrees of MAC significantly lower the mitral annular functional contribution to forward LV SV. Could this mean that severe MAC patients are better suited to purely annular-anchoring TMVR devices? Devices scaffolding the mitral annulus are likely to severely reduce or eliminate its function. However, severe MAC appears to have already preconditioned and inhibited the annular contribution to LV SV by ≈50%. Hence loss in LV SV from annular fixation is offset by the gains made in MR elimination, with an overall potential favorable net gain in forward LV SV. On the other hand, severe HFrEF patients with zero/little MAC and severe MR might not stand to benefit from annular fixation TMVR devices; rather annular sparing technologies might be preferred in such patients. These patients rely more on mitral annular functional contribution to LV SV, and mitral annular ‘freezing’ or ‘scaffolding’ may reduce LV SV by at least 50%. Unless the regurgitant volume from MR elimination is much larger than the loss of forwards LV SV, the net result in LV SV might not be favorable for the patient. Despite the echocardiogram in these patients showing no/mild residual MR post-TMVR, the thwarted effect on LV SV coupled with abnormal diastolic conditions at the LV base may ultimately result in a lack of clinical/functional improvement in patients. This may be a reason why M-TEER in such patients, despite residual MR, yields favorable clinical and functional outcomes as simply MR reduction (as opposed to total MR elimination) results in a net improvement in LV SV without deleterious effects on mitral annular function.
